# The High Price of Interrupted Follow‐Up: Catastrophic Progression of Homozygous Familial Hypercholesterolemia—A Case Report and Literature Review

**DOI:** 10.1002/ccr3.72028

**Published:** 2026-02-11

**Authors:** Parham Dastjerdi, Mahdieh Aghababaei, Reza Nikfar, Maryam Aghakouchakzadeh, Reza Mohseni‐Badalabadi, Kaveh Hosseini

**Affiliations:** ^1^ Tehran Heart Center, Cardiovascular Diseases Research Institute Tehran University of Medical Sciences Tehran Iran

**Keywords:** aortic stenosis, atherosclerotic cardiovascular disease, case report, homozygous familial hypercholesterolemia, lipid‐lowering therapy

## Abstract

Familial hypercholesterolemia (FH) is the most common monogenic lipid disorder, primarily resulting from mutations in LDLR, APOB, and PCSK9 genes. These mutations cause persistently high levels of low‐density lipoprotein cholesterol (LDL‐C), predisposing affected individuals to premature atherosclerotic cardiovascular disease (ASCVD). Homozygous FH (HoFH), a rare but severe form, manifests early in life with cutaneous xanthomas and accelerated coronary and aortic disease. Early diagnosis and aggressive, lifelong management are crucial, yet challenges remain, particularly when follow‐up is interrupted. We report the case of a 20‐year‐old female diagnosed with HoFH at age 13 after presenting with xanthomas. Initial evaluation revealed mild to moderate aortic stenosis and early coronary artery involvement. Genetic testing confirmed a homozygous LDLR mutation. Despite treatment with atorvastatin and evolocumab, partial lipid control was achieved, and follow‐up was disrupted during the COVID‐19 pandemic. At 20 years, she presented with worsening dyspnea, paroxysmal nocturnal dyspnea, and orthopnea. Advanced imaging documented severe heart failure with an ejection fraction of 20%, significant ventricular dilation, severe mitral regurgitation, and calcified aortic stenosis. Coronary angiography demonstrated critical coronary stenoses, while subsequent adjustments in her lipid‐lowering regimen, including rosuvastatin, ezetimibe, increased evolocumab dosing, and bempedoic acid, failed to stabilize her condition. Despite recommendations for surgical intervention, the patient's critical status precluded operative management, and she tragically died on the day of her scheduled follow‐up. This case underscores the aggressive natural history of HoFH and the dire consequences of interrupted follow‐up care. Early detection and sustained, multidisciplinary management are essential to mitigate rapid cardiovascular deterioration in HoFH patients. Consistent monitoring and prompt therapeutic adjustments remain pivotal in improving outcomes and reducing the high mortality risk associated with advanced aortic and coronary complications in these patients.

## Key Clinical Message

Homozygous familial hypercholesterolemia can progress to fatal, multi‐valvular and coronary disease in early adulthood despite maximal lipid‐lowering therapy, including statins, ezetimibe, PCSK9 inhibition, and bempedoic acid. Continuous follow‐up is indispensable to detect rapid deterioration and enable timely interventions that may alter the otherwise catastrophic course.

## 1 Introduction

Familial hypercholesterolemia (FH) is the most common monogenic disease, characterized by mutations in genes encoding proteins essential for lipoprotein metabolism—primarily the low‐density lipoprotein receptor (LDLR), apolipoprotein B (APOB), and PCSK9 [1, 2]. These genetic alterations result in lifelong elevated levels of low‐density lipoprotein cholesterol (LDL‐C), establishing the foundation for the early development of atherosclerotic cardiovascular disease (ASCVD) [2]. Homozygous familial hypercholesterolemia (HoFH), which affects approximately one in a million children globally, can lead to coronary heart disease (CHD) within the first decade of life, with many young patients experiencing myocardial infarctions before the age of 20 [3, 4, 5, 6]. In contrast, heterozygous familial hypercholesterolemia (HeFH) impacts about 1 in 250 individuals, potentially affecting an estimated 6.8–8.5 million children and adolescents worldwide [7]. Given the significant burden and early onset of disease, FH should be considered in patients under 20 years of age when LDL levels exceed 160 mg/dL [8].

Clinically, FH is manifested by elevated plasma LDL‐C concentrations, the presence of tendon xanthomas or corneal arcus, and a family history of premature CHD [9]. Diagnosis can be achieved through clinical assessment, detailed evaluation of personal and family history, or genetic testing [9]. Due to the high risk of premature ASCVD, particularly involving the coronary arteries, patients with FH require aggressive treatment [10, 11, 12]. Statins are the first‐line therapy, with ezetimibe as a second‐line option; PCSK9 inhibitors are employed less frequently [13]. In cases of HoFH, aortic supravalvular and/or valvular stenosis may also be observed and can be a primary cause of mortality [14, 15, 16].

In this case report and literature review, we present a detailed report of a young female with familial hypercholesterolemia whose clinical course exemplifies the challenges of managing advanced HoFH. The case highlights the interplay between a strong genetic predisposition and the progressive development of cardiovascular complications. By synthesizing the clinical evolution observed in our patient with current literature on the genetic basis, diagnostic challenges, and evolving treatment strategies in FH, we emphasize the critical need for early detection, continuous follow‐up, and aggressive, individualized management. This report aims to provide insights into the importance of proactive care and the potential consequences of disrupted monitoring, thereby informing clinicians about strategies to mitigate severe aortic and coronary complications in these patients. This case report was prepared in compliance with the CARE (CAse REport) guidelines to ensure completeness and transparency in reporting [17].

## 2 Case History / Examination

A 20‐year‐old female with a known history of FH diagnosed at age 13 was referred to our center for progressive worsening of exertional dyspnea, paroxysmal nocturnal dyspnea, and orthopnea over several months.

Her hypercholesterolemia had first manifested at 2 years of age with cutaneous xanthomas over the hands, elbows, and legs. At age 13, hospitalization for xanthoma progression led to diagnosis. At that time, transthoracic echocardiography (TTE) revealed mild to moderate aortic stenosis (pressure gradient [PG] 46 mmHg, mean gradient [MG] 26 mmHg), left ventricular ejection fraction (LVEF) 60%, and mild mitral regurgitation (MR). Coronary computed tomography angiography (CTA) showed mild coronary artery stenosis. Genetic testing confirmed a homozygous pathogenic LDLR mutation (c.389C>G; rs879254509). Her lipid profile was markedly abnormal: total cholesterol 818 mg/dL, LDL‐C 710 mg/dL, HDL‐C 56 mg/dL, triglycerides 272 mg/dL.

Initial treatment included atorvastatin 40 mg daily and two courses of evolocumab (three subcutaneous injections of 140 mg each), which reduced total cholesterol to 586 mg/dL and LDL‐C to 457 mg/dL, with minimal change in triglycerides. She was discharged on atorvastatin, propranolol, captopril, and aspirin. Follow‐up was interrupted during the COVID‐19 pandemic.

**FIGURE 1:**
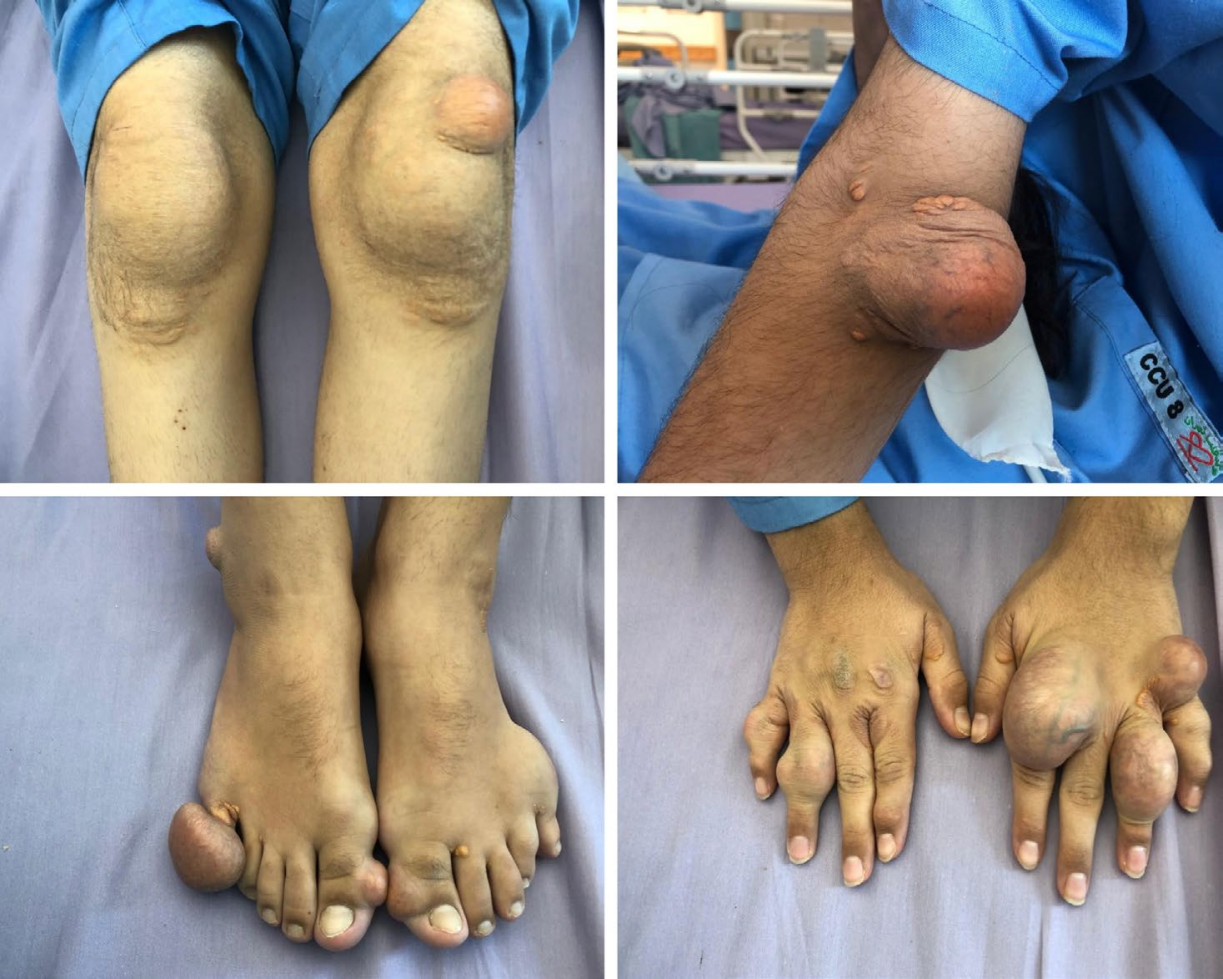
Extensive cutaneous xanthomas on the patient's extremities, including the knees, elbows, feet, and hands.

**FIGURE 2:**
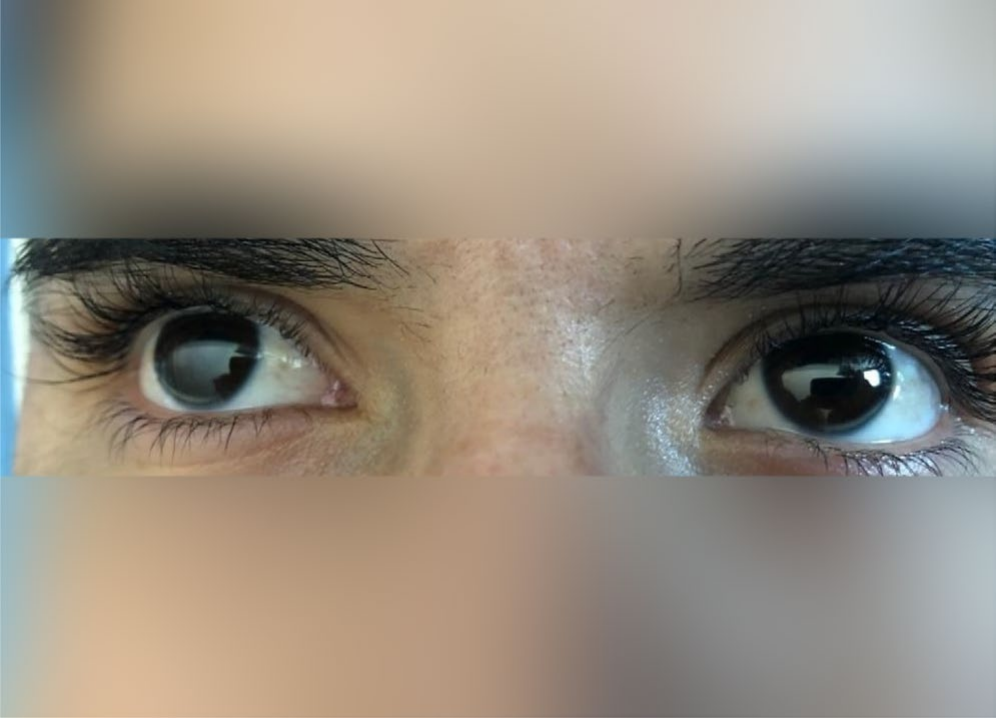
Bilateral corneal arcus in both eyes of the patient.

On her current presentation at age 20, she appeared dyspneic at rest. Physical examination revealed extensive cutaneous xanthomas (Figure 1) and bilateral corneal arcus (Figure 2).

## 3 Differential Diagnosis, Investigations, and Treatment

Although the patient carried a confirmed diagnosis of HoFH, the acute presentation with worsening dyspnea, orthopnea, and paroxysmal nocturnal dyspnea warranted consideration of other potential causes of decompensation. In the context of severe dyslipidemia and prior aortic valve disease, differentials are included in Table 1.

**TABLE 1:** Differential diagnoses and pathophysiologic mechanisms considered in this patient's acute decompensation.

Category	Differential diagnosis	Mechanism/clinical relevance
Valvular heart disease	Progression of aortic stenosis (valvular or supravalvular)	Worsening outflow obstruction leading to increased left ventricular pressure and heart failure symptoms
Ischemic heart disease	Progression of coronary artery disease with ischemic cardiomyopathy	Myocardial ischemia and left ventricular dysfunction causing reduced cardiac output
Secondary valvular disease	Severe mitral regurgitation secondary to ventricular remodeling	Left ventricular dilation and dysfunction leading to functional mitral regurgitation and volume overload
Non‐cardiac causes	Anemia, pulmonary embolism, or severe pulmonary hypertension	Reduced oxygen delivery or increased pulmonary vascular resistance mimicking or worsening heart failure

**FIGURE 3:**
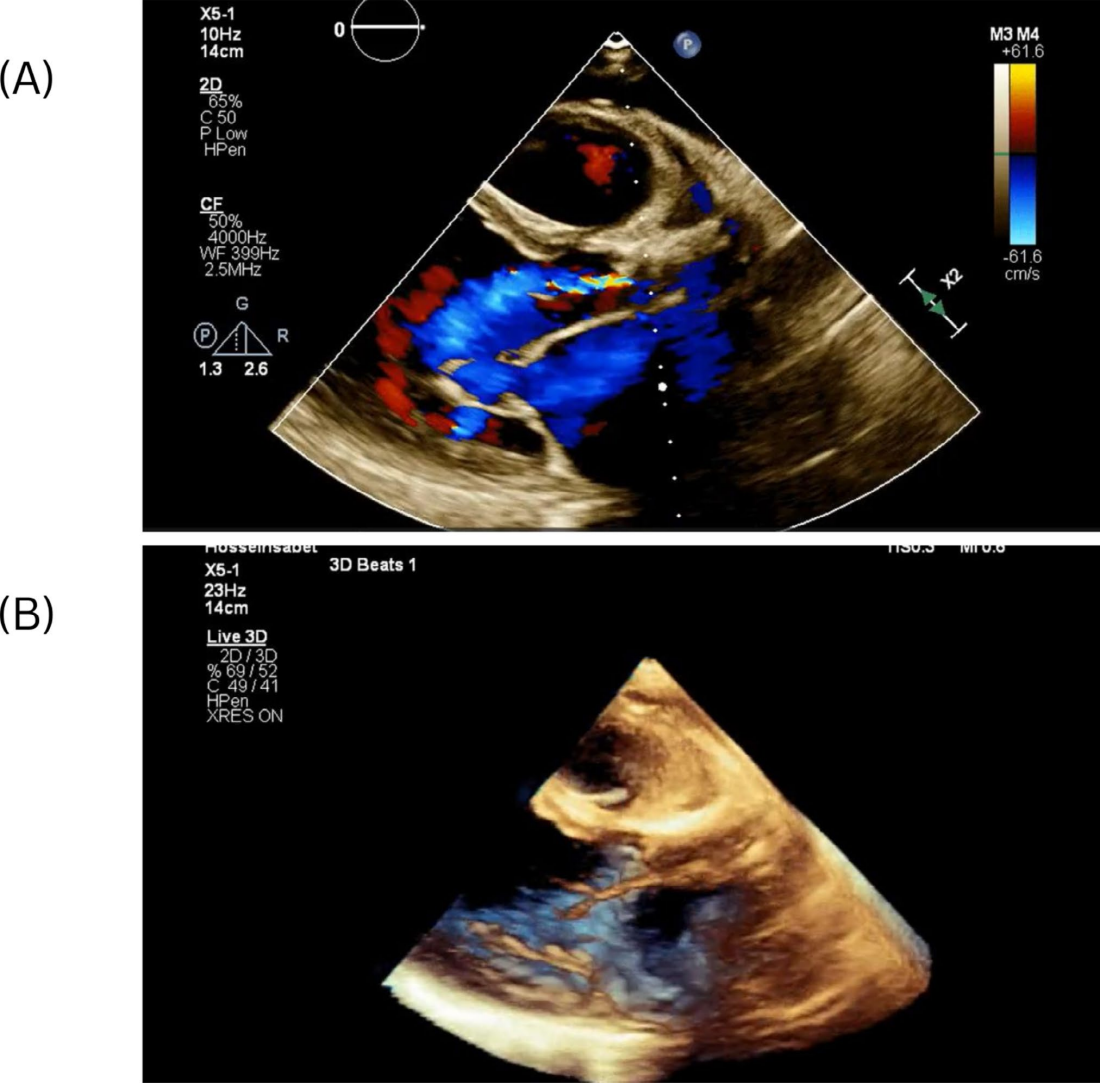
(A) 2D color Doppler echocardiography of the parasternal view showing supravalvular AS. (B) 3D echocardiography of the parasternal view demonstrating the same supravalvular AS.

**FIGURE 4:**
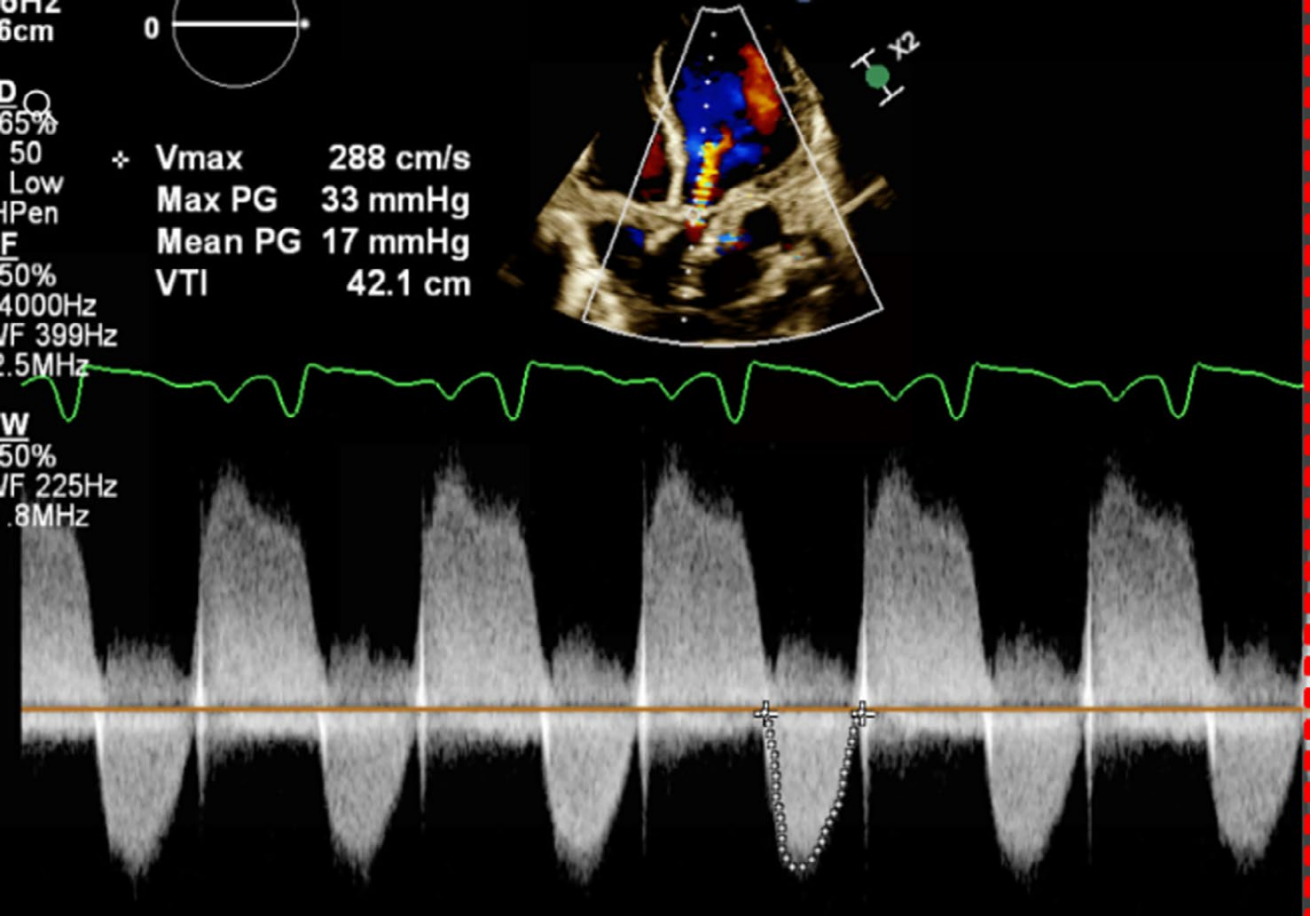
CW Doppler showing severe AS in the 4‐chamber view.

2D and 3D echocardiography with continuous wave (CW) Doppler revealed severe left ventricular systolic dysfunction (EF 20%), marked ventricular dilation, severe MR, and calcified aortic stenosis (PG 44 mmHg, MG 26 mmHg) with an annulus diameter of 18–19 mm. Figure 3 presents the 2D and 3D color Doppler echocardiography cuts of the parasternal view of the patient, showing the supravalvular aortic stenosis (AS). Figure 4 demonstrates the severe AS and aortic insufficiency (AI) using CW color Doppler in the 4‐chamber view.

Coronary angiography demonstrated critical ostio‐proximal left main (LM) stenosis, mild proximal left anterior descending (LAD) stenosis, moderate proximal left circumflex (LCX) stenosis, and significant ostio‐proximal and mid‐right coronary artery (RCA) stenosis. Gastroenterology consultation and liver biopsy confirmed stage F2 hepatic fibrosis. Admission lipid profile showed: total cholesterol 539 mg/dL, LDL‐C 351 mg/dL, HDL‐C 27 mg/dL, triglycerides 392 mg/dL.

These findings prompted the initiation of further lipid‐lowering therapy. Following a consultation with a clinical pharmacist, her lipid‐lowering regimen was adjusted. Rosuvastatin 40 mg daily was substituted for atorvastatin 40 mg daily, ezetimibe 10 mg daily was added, and the patient was prescribed evolocumab injection 420 mg per month. Heart failure management was also optimized, including dapagliflozin, metoprolol succinate, furosemide, and spironolactone.

**FIGURE 5:**
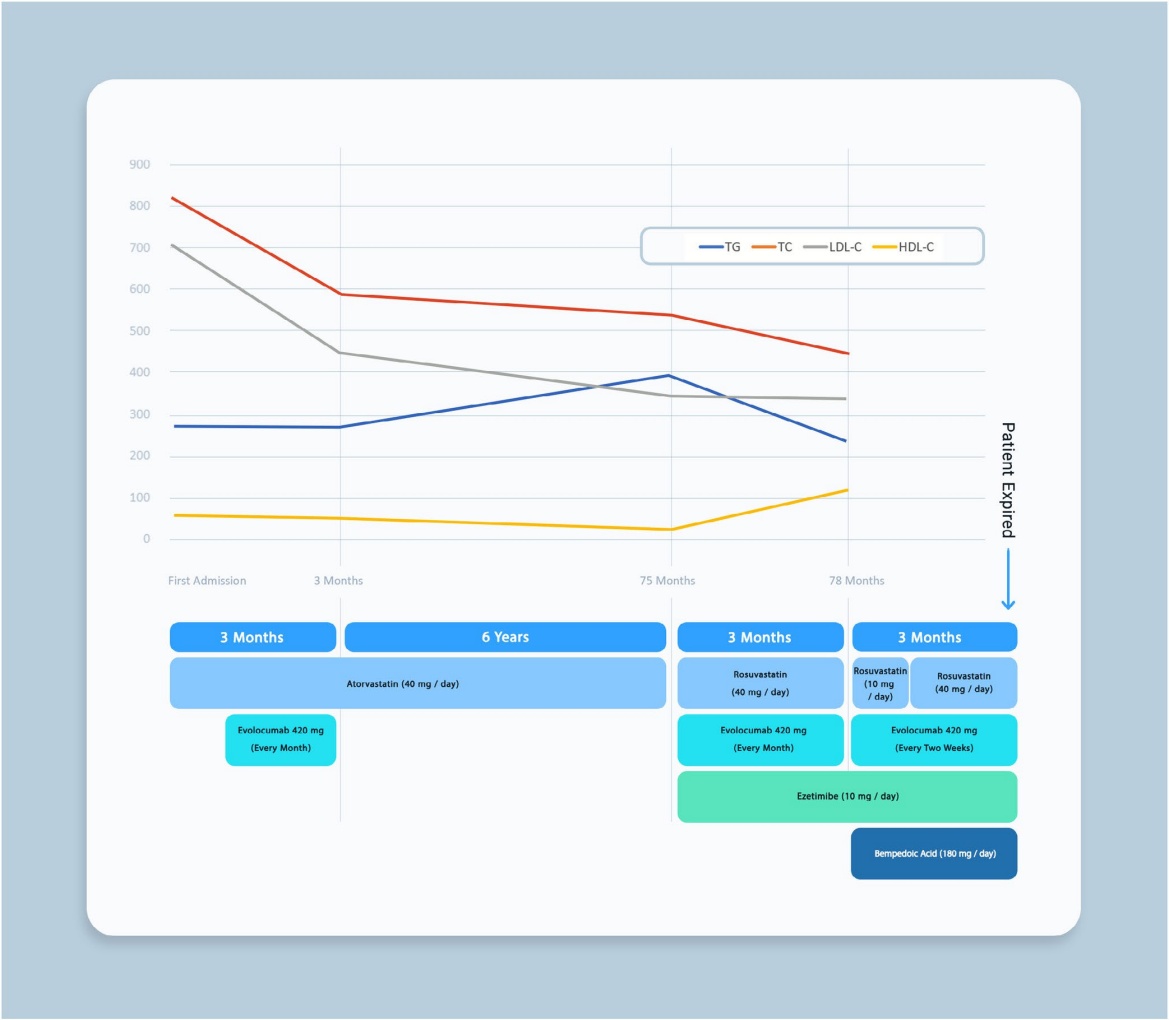
Timeline of lipid‐lowering therapy adjustments and corresponding lipid profile changes.

After 3 months, the patient's lipid profile was as follows: triglycerides at 241 mg/dL, total cholesterol at 456 mg/dL, LDL‐C at 341 mg/dL, and HDL‐C at 115 mg/dL. Due to elevated liver function tests, the rosuvastatin dosage was reduced from 40 mg daily to 10 mg daily. Additionally, given the inadequate lipid control, the evolocumab dosage was increased from 420 mg monthly to 420 mg every 2 weeks. Once liver function tests normalized, the rosuvastatin dosage was gradually restored to 40 mg daily, and the patient was initiated on bempedoic acid 180 mg daily. The sequential changes in lipid‐lowering therapy and corresponding lipid profile fluctuations are illustrated in Figure 5, highlighting the patient's response over time.

Given the severity of her disease, the medical team initially recommended aortic valve replacement (AVR) and coronary artery bypass grafting (CABG) as the primary treatment options. However, due to the high surgical risk, the decision was made to postpone surgery and assess the patient's response to the final adjustments in lipid‐lowering therapy, including the initiation of bempedoic acid.

## 4 Outcome and Follow‐Up

Before re‐evaluation and planned follow‐up laboratory testing, the patient suffered sudden death on the day of her scheduled appointment. No further interventions, such as percutaneous coronary intervention (PCI), transcatheter aortic valve implantation (TAVI), or consideration of combined heart–liver transplantation, could be undertaken.

## 5 Discussion

FH is an autosomal dominant genetic disorder marked by significantly elevated LDL‐C levels and an increased risk of premature ASCVD. It results primarily from mutations in the LDLR gene, which impairs LDL‐C clearance. Additional mutations in APOB and PCSK9 contribute to FH by reducing LDL receptor binding affinity and enhancing receptor degradation, respectively [18]. In heterozygous FH (HeFH), a single mutated allele leads to a twofold rise in LDL‐C, whereas homozygous FH (HoFH)—caused by mutations in both alleles—can increase LDL‐C levels to more than five times normal values [19, 20]. Over 1200 LDLR variants have been identified, with specific mutations in APOB (e.g., R3500Q) and gain‐of‐function mutations in PCSK9 further worsening receptor function [18, 21, 22].

HoFH presents with an aggressive phenotype. When LDL receptor activity is < 2%, prognosis is poor, with many patients not surviving beyond their twenties without intervention. Even partial receptor activity (2%–25%) can lead to severe coronary and aortic valve disease by age 30 [19]. Early manifestations include cutaneous and interdigital xanthomas in childhood, and LDL‐C levels often exceed 500 mg/dL in untreated individuals [20, 23]. The progression of the disease is intimately linked to both the magnitude and duration of LDL‐C elevation, with myocardial infarctions and sudden cardiac death reported in children as young as 4 years old [4, 24, 25]. In addition to coronary complications, many HoFH patients suffer from supravalvular and aortic valve stenosis, often necessitating surgical intervention later in life [26, 27, 28]. Lipid accumulation in the cornea, which manifests as corneal arcus, further underscores the severity of the condition [29]. Our patient demonstrated several of these classic features. From an early age, she developed cutaneous xanthomas on her extremities—a hallmark of the disease. By her early twenties, bilateral corneal arcus was evident, reflecting profound lipid accumulation. Imaging studies confirmed advanced cardiovascular involvement, revealing severe aortic stenosis, significant coronary artery disease with critical ostio‐proximal stenoses, and supravalvular aortic stenosis.

Timely diagnosis of HoFH is essential to initiate aggressive treatment strategies and improve long‐term outcomes. Clinically, HoFH is suspected in individuals presenting with markedly elevated LDL‐C levels—often exceeding 500 mg/dL (13 mmol/L)—in conjunction with a family history of premature cardiovascular events or the presence of physical stigmata such as xanthomas emerging in early childhood [30]. Genetic testing plays a pivotal role in confirming the diagnosis, as it can identify pathogenic variants in genes like LDLR, APOB, or PCSK9, thereby differentiating HoFH from severe forms of HeFH [30]. In clinical practice, several validated diagnostic frameworks are available, including the MedPed criteria [31], the Dutch Lipid Clinic Network scoring system [32], the Simon Broome Register criteria [33], and population‐specific guidelines such as the Japanese FH criteria [34], with genetic testing serving as a confirmatory tool when available. In resource‐limited settings or when genetic testing is not accessible, diagnosis is primarily based on these clinical tools together: untreated LDL‐C levels above 500 mg/dL or treated levels above 300 mg/dL, particularly when coupled with early‐onset xanthomas or a known family history of hypercholesterolemia [30]. Supportive imaging studies—such as carotid intima‐media thickness (CIMT), coronary artery calcium (CAC) scoring, and echocardiography—often reveal significant atherosclerosis and aortic valve disease, further aiding in the diagnostic process [19]. However, the overlap in phenotypic expression and the variability in genetic mutations necessitate an interdisciplinary approach, involving genetic counseling, lipidology, and cardiology, to confirm the diagnosis and design an appropriate treatment plan.

The management of HoFH is complex and requires an individualized, stepwise approach depending on disease severity and treatment response. High‐intensity statins (simvastatin, atorvastatin, or rosuvastatin) form the cornerstone of therapy, though they are often insufficient due to limited LDL receptor function. Therefore, combination therapy with ezetimibe, bile acid sequestrants, and fibrates is frequently required [20]. In our case, despite maximally tolerated statins and ezetimibe, LDL‐C levels remained markedly elevated, prompting the initiation of evolocumab. PCSK9 inhibitors like evolocumab and alirocumab have been shown to lower LDL‐C by approximately 30% in patients with receptor‐defective mutations, as evidenced by the TESLA Part B trial [13, 35].

More recently, novel agents such as lomitapide, a microsomal triglyceride transfer protein (MTP) inhibitor, and evinacumab, an angiopoietin‐like protein 3 (ANGPTL3) inhibitor, have demonstrated LDL‐C reductions of up to 50% and have become valuable options for refractory cases [36, 37, 38]. The ELIPSE HoFH trial and subsequent pediatric studies have confirmed evinacumab's efficacy, with LDL‐C reductions approaching 48%–49% even in patients already on maximum lipid‐lowering therapy [38, 39]. Unfortunately, these therapies were not available in our country, limiting the options for our patient. More recently, bempedoic acid, an oral adenosine triphosphate citrate lyase inhibitor, has shown promise by providing an additional 15%–20% reduction in LDL‐C in patients with ASCVD or HeFH on maximal statin therapy, although its efficacy in HoFH remains to be fully established [40, 41, 42]. In our patient, bempedoic acid was introduced as an adjunct after prior therapies failed to achieve optimal control; however, she passed away before the clinical impact of this addition could be fully evaluated. When pharmacotherapy fails to achieve target LDL‐C levels, lipoprotein apheresis becomes a crucial option, typically achieving 50%–70% LDL‐C reduction per session [43, 44]. In our patient's case, we were considering lipoprotein apheresis as the next step if bempedoic acid proved insufficient. Unfortunately, due to her untimely death, this intervention could not be pursued. In advanced cases with progressive, treatment‐resistant atherosclerosis, liver transplantation may be considered as a last resort, providing functional LDL receptors but introducing the burden of lifelong immunosuppression and procedural risks [14].

The management of HoFH, particularly when complicated by CAD and aortic stenosis, remains exceptionally challenging. Patients with HoFH often experience aggressive cardiovascular disease that necessitates high‐risk, complex surgical interventions. For instance, a reported case of two cousins with HoFH required intricate procedures including the Bentall operation, mitral valve replacement, and CABG due to extensive atherosclerotic involvement [45]. Long‐term management also demands continuous monitoring and specialized care. A longitudinal study of 39 HoFH patients revealed that, despite early and aggressive lipid‐lowering therapy, many progressed to severe CAD and aortic valve disease, ultimately necessitating surgical intervention [46]. Furthermore, delayed diagnosis can have catastrophic outcomes. A case report from Egypt detailed two young women, aged 21 and 27, who were diagnosed with HoFH only after the onset of severe vascular complications, including aortic stenosis and multi‐vessel CAD—with one patient dying at the age of 21 [47]. Table 2 provides details on eight cases of complicated HoFH patients.

**TABLE 2:** Literature review table.

	Summary	Treatment plan	Outcome	Follow‐up
Kolovou‐2006 [48]	**A 13‐year‐old Greek boy with FH** presented with severe dyslipidemia, characterized by high cholesterol levels, xanthomas supravalvular AS, and carotid artery stenosis.	**Medical Therapy:** At first a combination of statins and cholestyramine. Then, ezetimibe was added. **LDL Apheresis:** LDL apheresis every 10 days.	**Lipid Profile Improvement:** Reductions in total cholesterol, LDL‐C, and triglycerides, and relatively unchanged HDL‐C **Xanthomas Regression:** Significant regression of tuberous xanthomas on the knees and elbows. **Clinical Progression:** surgery for supravalvular AS.	NA
Ozumi‐2005 [49]	**A 64‐year‐old woman with FH** with chest pain and dyspnea. She had AS and supravalvular AS.	**Aortic Valve Replacement:** Aortic valve replacement to address the AS. **Lipid‐Lowering Therapy:** A combination of statin (simvastatin) and cholestyramine. **LDL Apheresis:** LDL apheresis was also initiated. **Beta‐blocker:** Metoprolol was prescribed to control residual AS.	**Angina Resolution:** The patient's angina symptoms resolved after TAVR. **Improved Lipid Profile:** Significant reduction of LDL‐C level through the combination of medications and apheresis. **On‐going Management:** The patient required ongoing treatment to prevent complications.	NA
Rasheed‐2024 [50]	**A 16‐year‐old female with FH** presented with chest pain and dyspnea. She had a history of cutaneous xanthomas and was found to have severe AS, CAD, and left ventricular dysfunction.	**PCI:** The patient underwent PCI for LAD and right coronary arteries. **Medical Therapy:** A high‐dose statin and ezetimibe	**Improved Cardiac Function:** Following treatment plans the patient's left ventricular systolic function improved, and mitral regurgitation was reduced. **Symptom Relief:** The patient experienced improvement in her symptoms.	Follow‐up after 1 month indicated asymptomatic status and regular clinical monitoring.
Sahiner‐2016 [51]	**A 59‐year‐old woman with FH and a history of coronary artery bypass grafting** presented with chest discomfort and dyspnea. She had severe AS and a porcelain aorta, which precluded surgical valve replacement.	**PCI:** The patient underwent PCI in the RCA‐OM saphenous vein graft. **TAVI:** TAVI was conducted for AS. **Pre‐Procedural Evaluation:** TEE and MSCT were performed to assess the aortic anatomy and identify potential complications.	**Successful TAVI:** TAVI successfully reduced the aortic stenosis gradient and left minor regurgitation. **Improved Symptoms:** After TAVI, the patient's chest pain and dyspnea have improved.	NA
Alenizi‐2020 [45]	**A 39‐year‐old male diagnosed with HoFH at age 14**, presented with symptoms of heart failure and significant cardiovascular complications, including SVAS and CAD.	**Statin Therapy:** At first Simvastatin was given to the patient. **LDL Apheresis:** The patient started LDL plasma apheresis at 17. **Surgical Interventions:** The patient had the Bentall operation, CABG surgery, and mechanical mitral valve replacement. **Medications:** He takes rosuvastatin, evolocumab, ezetimibe, aspirin, atenolol, and warfarin.	**Post‐Surgery Stability:** The patient's condition stabilized after surgeries. **Functional Mechanical Valves:** Mechanical valves functioned well	Regular LDL apheresis and echocardiography. His general condition remained stable with no complication
Alkhateeb‐2013 [47]	**Two Egyptian women with HoFH were diagnosed late, after presenting with severe vascular complications**. Recurrent angina and syncope plagued the first patient, a 21‐year‐old woman with xanthomas misdiagnosed as lipomas. She died before surgery from significant AS and CAD. In the second patient, aged 27, a forearm amputation was necessary due to left brachial artery occlusion. PCI was performed for mid‐right coronary artery and left anterior descending artery.	**First Patient:** Initially given aspirin, nitrates, bisoprolol, and atorvastatin. After myalgia, atorvastatin was lowered and replaced with simvastatin/ezetimibe. A TAVR and CABG were planned but never executed. **Second Patient:** Treated with atorvastatin, dual antiplatelet therapy, and bisoprolol. She underwent PCI with drug‐eluting stents.	**First Patient:** Developed xanthomas, AS, and multi‐vessel CAD. She died after surgical excision of xanthomas. **Second Patient:** Successfully underwent PCI and delivered a healthy baby but later developed recurrent angina and required further stenting.	**First Patient:** NA. **Second Patient:** Asymptomatic for two years after PCI, but later experienced recurrent angina and is scheduled for another PCI.
Chowdhury‐2020 [52]	**A 19‐year‐old male from India with HoFH** The patient had bilateral corneal arcus, xanthelasma on eyelids, and cutaneous xanthomas on arms, elbows, wrists, knees, and buttocks. Echocardiography and CT showed supravalvular and valvular AS. Aortic root enlargement and mechanical AVR were performed due to increasing angina.	**Medical Management:** The patient was treated with statins, beta‐blockers, and aspirin prior to surgery **Surgical Intervention:** The patient underwent Nick's procedure and TAVR. The aortic annular defect was repaired using autologous pericardium.	**Post‐Surgery Recovery:** His surgical recovery was uncomplicated. No supravalvular AS remained, and exercise tolerance improved. **Histological Findings:** Atheromatous plaque with cholesterol clefts and lipid‐laden macrophages was found in the excised aortic tissue.	At 12 months, the patient had better exercise tolerance, no daily angina, and a stable mechanical valve with a 10 mmHg mean trans‐prosthetic gradient.
Davoodabadi‐2022 [53]	**A 15‐year‐old boy with HoFH** exertional dyspnea and chest discomfort progressed. Hyperlipidemia and premature cardiovascular mortality were in his family. After abrupt non‐STEMI, the patient developed HF and cardiogenic shock. Echocardiography and coronary angiography demonstrated substantial left ventricular dysfunction, valvular and supravalvular AS, and two‐vessel left main CAD.	**Medical Therapy:** The patient was treated with aspirin, rosuvastatin, ezetimibe, intravenous heparin, furosemide, and norepinephrine for stabilization. **Surgical Intervention (Planned):** CABG, AVR, and mitral valve repair were planned but not completed due to patient deterioration before surgery.	**Clinical Deterioration: T**he patient developed worsening tachycardia, hypotension, and HF before surgery, leading to death.	NA

Abbreviations: AS, aortic stenosis; AVR, aortic valve replacement; CABG, coronary artery bypass grafting; CAD, coronary artery disease; CT, computed tomography; FH, Familial Hypercholesterolemia; HDL‐C, high‐density lipoprotein cholesterol; HF, heart failure; HoFH, homozygous familial hypercholesterolemia; LAD, left anterior descending artery; LDL, low‐density lipoprotein; LDL‐C, low‐density lipoprotein cholesterol; MSCT, multislice computed tomography; NA, not available; PCI, percutaneous coronary intervention; RCA‐OM, right coronary artery‐obtuse marginal; SVAS, supravalvular aortic stenosis; TAVI, transcatheter aortic valve implantation; TAVR, transcatheter aortic valve replacement; TEE, transesophageal echocardiography.

## 6 Conclusion

Lifelong monitoring of HoFH patients is essential to ensure timely treatment adjustments and make critical decisions as new manifestations arise. In the case presented, the patient's discontinuation of follow‐up visits due to the COVID‐19 pandemic had severe consequences. The absence of regular monitoring meant that necessary adjustments to medications and treatments were not made, and significant changes in EF and other clinical parameters went unnoticed by the care team. Consequently, the patient returned to our center with significantly progressed disease and in critical condition. This case underscores the vital importance of consistent follow‐up and proactive management in preventing the rapid deterioration often seen in HoFH patients.

## Author Contributions

**Parham Dastjerdi:** conceptualization, investigation, methodology, project administration, supervision, visualization, writing – original draft, writing – review and editing. **Mahdieh Aghababaei:** conceptualization, project administration, supervision. **Reza Nikfar:** investigation, writing – original draft. **Maryam Aghakouchakzadeh:** conceptualization, investigation, methodology, validation, writing – review and editing. **Reza Mohseni‐Badalabadi:** supervision, validation. **Kaveh Hosseini:** conceptualization, data curation, investigation, methodology, project administration, supervision, validation, writing – review and editing.

## Funding

The authors have nothing to report.

## Ethics Statement

The authors have nothing to report.

## Consent

The patient and their family provided written informed consent for the publication of this case report.

## Conflicts of Interest

The authors declare no conflicts of interest.

## Data Availability

The authors have nothing to report.
